# Pregnancy in Patients with Moderate and Highly Complex Congenital Heart Disease

**DOI:** 10.3390/healthcare11111592

**Published:** 2023-05-30

**Authors:** Mario Panebianco, Marco Alfonso Perrone, Maria Giulia Gagliardi, Lorenzo Galletti, Pier Paolo Bassareo

**Affiliations:** 1 Department of Cardiac Surgery, Cardiology, Heart and Lung Transplantation Bambino Gesu’ Children’s Hospital, IRCCS, 71013 Rome, Italy; 2 Division of Cardiology and CardioLab, Department of Clinical Sciences and Translational Medicine, University of Rome Tor Vergata, 00133 Rome, Italy; 3 School of Medicine, University College of Dublin, Mater Misericordiae University Hospital and Children’s Health Ireland Crumlin, D07 R2WY Dublin, Ireland

**Keywords:** congenital heart disease, pregnancy, GUCH, ACHD, risk stratification

## Abstract

Although not completely devoid of risk, pregnancy can be managed in virtually all patients affected by even the most complex forms of congenital heart disease. It is not however advisable in patients with any form of pulmonary arterial hypertension. Pregnancy is even manageable in patients with univentricular heart converted to Fontan circulation. A personalised risk stratification should be performed, and patients affected by advanced NYHA functional class appropriately warned of the potential risks. In this setting, metabolomics might represent a novel tool for use in conducting personalised risk stratification. All pregnancies, particularly those at higher risk, should be managed in a tertiary care centre capable of providing the necessary assistance to both the mother and infant. With a few rare exceptions, vaginal delivery is to be preferred over caesarean section due to the lower degree of maternal and foetal complications. The desire for motherhood, at times extreme in women with congenital heart disease, may often be accomplished, thus providing a ray of hope in the lives of these patients.

## 1. Introduction

A significantly higher number of cases of congenital heart disease (CHD) is observed in the adult population (adult congenital heart disease, ACHD) when compared to the number of live paediatric subjects affected by the same condition. Indeed, early diagnosis and progresses made in the field of interventional and surgical treatment have enabled more than 90% of CHD patients to reach adulthood [1]. A large percentage of these ACHD patients are women and, reaching childbearing age, may wish to experience motherhood [2].

The aim of this narrative review is to assess the risk and the management of pregnancy in moderate or great complexity ACHD patients.

The prevalence of CHD worldwide is approximately 9/1000 neonates, with some geographical variations [3]. A slightly lower prevalence (particularly of complex CHD) is observed in industrialised countries due to the widespread availability of foetal screening and prenatal diagnosis; whilst in developing countries (in which these are less available or at times totally lacking), a higher prevalence of infants born with CHD is observed [1]. As stated previously, the ACHD population also includes women of childbearing age affected by complex congenital heart disease and undergoing palliative treatment, who hope to become mothers in the future. Pregnancy in these women constitutes a high-risk factor, with an estimated cardiovascular risk of complications of approximately 1–4% [4,5]. This increased risk however is not produced only by pregnancy, with the presence of residual alterations of cardiac function, including major valve defects, systolic and diastolic dysfunction, cyanosis and varying degrees of pulmonary hypertension all acting as contributing factors.

To investigate why pregnancy is so poorly tolerated in this population, a series of physiological changes that occur during pregnancy should be taken into account [6,7].

## 2. Adaptation of the Cardiovascular System during Pregnancy

As early as the sixth week of gestation, the primordial heart starts to beat. Likewise, the increase in blood volume manifested throughout pregnancy is first detected in the early weeks of pregnancy and increases rapidly up until the fourth/fifth month of gestation, subsequently progressing at a slower rate [6,7].

Concomitantly, systemic vascular resistance decreases by approximately 30% compared to baseline, due to both the effect of pregnancy hormones and subsequent development of the placenta. This in turn determines reduced vascular tone with consequent decrease in systemic blood pressure (BP) [6,7].

This vasodilation is compensated for by an approx. 30–50% increased cardiac output (CO) compared to baseline. This increased output is the result of an increase in both stroke volume and heart rate, with the latter occurring largely during the third trimester. These hemodynamic changes place an additional burden on the heart with increased end-systolic and end-diastolic ventricular volumes. At the end of pregnancy, labour and delivery are both seen as critical events due to the presence of an additional rise in cardiac output and increased oxygen demand. Uterine contractions each produce an approx. 500 cc increase in circulating blood volume. During delivery, a sudden increase in systemic vascular resistance elicited by placental expulsion determines an increment in ventricular filling pressure. Baseline conditions are normally restored within 2–6 weeks of vaginal delivery, or longer in the case of caesarean section. The heart then continues to be subjected to a higher burden following delivery as, paradoxically, although uterine compression on the inferior vena cava lessens, an increased systemic venous return to the heart is manifested [6,7].

Pregnancy is intrinsically associated with a state of hypercoagulability, a factor to not underestimate in the presence of a series of conditions (e.g., pulmonary arterial hypertension) [8].

It is evident therefore that the cardiovascular system of a patient affected by depressed cardiac function, increased pulmonary pressure or single ventricle circulation will be faced with a series of major challenges as pregnancy commences.

Nevertheless, as a general rule, vaginal delivery is the preferred option, with elective caesarean section limited to women affected by ventricular dysfunction and advanced NYHA functional status [9]. This is because in the trade-off with caesarean section, complications (haemorrhage, infection, haemodynamic disturbancies, coagulation disorders) are less frequent [9].

**Figure 1:**
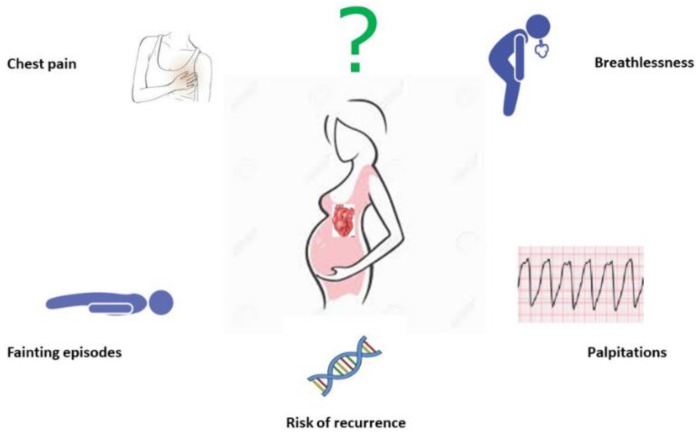
Major cardiac complications include onset of arrhythmias, heart failure, fainting episodes, chest pain, and risk of recurrence.

Major cardiac complications during pregnancy include onset of arrhythmias, in particular ventricular arrhythmia, heart failure, fainting episodes, chest pain and thromboembolic events (notably in women with mechanical heart valves) [7] (Figure 1).

Patients with Fontan circulation and cyanotic heart disease have been found to be at higher risk of spontaneous or elective miscarriage [10].

A mortality rate of up to 4% has been reported for infants born to mothers affected by congenital heart disease [11,12].

### 2.1. The ROPAC Registry (Registry of Pregnancy and Cardiac Disease)

The ROPAC registry is the main source of information for issues relating to pregnancy and heart disease. Published for the first time in 2007, the registry is kept continuously updated with data deriving from numerous hospitals worldwide (the majority of which European), and currently includes data from approx. 6000 pregnant women affected mainly by congenital (57%) and valvular heart disease (29%) [13].

The ROPAC registry highlighted the highest mortality rate in patients with pulmonary hypertension (+0.6% compared to the general population). Heart failure was diagnosed in 11% of the population, whilst arrhythmias were present in 2% of cases. The main risk factors for onset of maternal complications during pregnancy comprised pre-existing heart failure, NYHA functional class > II, systemic ejection fraction < 40%, mWHO functional class 4 and use of anticoagulants [13].

Accordingly, risk stratification represents an important tool aimed at limiting potential maternal and foetal complications.

### 2.2. Risk Stratification

The risk of onset of pregnancy-related cardiac complications may be predicted using a series of scoring systems such as CARPREG (CARdiac disease in PREGnancy) and ZAHARA. To date, however, higher-risk populations (e.g., patients affected by pulmonary arterial hypertension or aortic dilation) have been under-represented in both systems; therefore, the currently recommended and most widely used scoring system is the World Health Organisation (mWHO) risk scale [7,14], which divides patients into classes of risk based on their initial pathology, comorbidity and maternal risk factors.

Patients are thus classified as: very low risk (class I), low to moderate risk (class II), high risk (class III), in which pregnancy is not advised, and lastly, extremely high risk (class IV), in which pregnancy is contraindicated [14].

The first group (mWHO I) comprises minor corrected heart defects (ASD, VSD, PDA, partial pulmonary venous returns), mild pulmonary stenosis, minor valve defects and isolated atrial and ventricular extra-systoles.

The second group (mWHO II) includes patients with minor uncorrected defects (ASD e VSD) and corrected tetralogy of Fallot, the majority of supraventricular and ventricular arrhythmias and Turner’s syndrome without aortic dilation.

The intermediate group (mWHO II–III) comprises patients with mild ventricular dysfunction (EF > 45%), hypertrophic cardiomyopathy, mild mitral valve stenosis, moderate aortic stenosis, Marfan syndrome without aortic dilatation, aorta < 45 mm (in aortic disease associated with bicuspid aortic valve), repaired coarctation and repaired atrioventricular canal.

The third group (mWHO III) is made up of patients affected by moderate ventricular dysfunction, EF 30–45%, previous peripartum cardiomyopathy with no residual impairment of systolic function, patients with a mechanical valve, patients with Fontan circulation, systemic right ventricle with conserved or slightly reduced systolic function, cyanotic heart disease (unrepaired), moderate stenosis of the mitral valve, severe asymptomatic aortic stenosis, moderate aortic dilatation 40–45 mm in patients with Marfan syndrome, aortic dilatation 45–50 mm in patients with bicuspid aortic valve, patients with Turner’s syndrome and ASI (aortic size index) of 20–25 mm/m^2^; patients with tetralogy of Fallot and aortic diameter < 50 mm.

The last group (mWHO IV) includes patients with any form of pulmonary arterial hypertension, severe systemic ventricular dysfunction (left ventricle ejection fraction < 30%, New York Heart Association functional class III–IV), previous peripartum cardiomyopathy with residual ventricular dysfunction, severe stenosis of the mitral valve, severe symptomatic aortic stenosis, patients with moderate or severe systemic right ventricle dysfunction, Marfan syndrome with aortic dilatation > 45 mm, aortic dilatation > 50 mm in the context of aortic disease associated with bicuspid aortic valve, patients with Turner’s syndrome and ASI > 25 mm/m^2^; patients with tetralogy of Fallot and ascending aorta diameter > 50 mm, severe aortic coarctation, patients with complex Fontan circulation [14].

Maternal mortality risk increases gradually between the first and last group patient groups, with a frequency of maternal cardiac events ranging from 2.5 to 5% amongst patients in the first group to 40–100% in patients from the 4th group [14].

Pre-conception counselling should be provided to all women intending to initiate pregnancy, in particular patients affected by complex congenital heart disease. Counselling should be administered by either a cardiologist with expertise in the field of congenital adult heart disease or a gynaecologist with expertise in the specific field (multidisciplinary team) [15].

Pregnant women with NYHA functional class I may not need to be managed in a tertiary care hospital, whilst patients with NYHA functional class II or higher should always receive care in this type of setting. Delivery should take place in a hospital setting equipped to provide adequate support to both mothers and infants [14].

A pathophysiological classification of CHD [16] with related management pre-, during, and post-pregnancy is summarised in Table 1.

Below, the authors evaluate pregnancy management in the presence of a series of major highly or moderately complex heart diseases.

### 2.3. Tetralogy of Fallot

Tetralogy of Fallot (TOF) is a common cyanotic congenital heart disease accounting for approx. 5–6% of all congenital heart diseases. The American Heart Association classifies TOF as a moderately complex heart disease [17,18].

In the presence of TOF, gestational risks are associated with potential surgical repair, presence of residual defects and patient’s overall clinical status. Healthcare staff involved in patient care should be fully trained to recognise the different scenarios that may be presented. In the absence of significant residual defects or post-repair complications and presence of normal ventricular function pregnancy is considered low risk [19].

Patients subjected to transannular patch repair tend to develop severe pulmonary insufficiency. Consequently, the right ventricle adapts to this continual volume overload by dilating (Frank-Starling law). Significantly dilated and malfunctioning right ventricles do not tolerate pregnancy well, and whilst pulmonary valve failure per se is not a serious problem, it does however add an additional volumetric burden beyond the physiological increase present in pregnancy. Prior to programming a pregnancy, pulmonary insufficiency should be corrected, preferably by means of percutaneous biological valve implantation, where possible, or alternatively, surgery. Although the indication for correction is clear in symptomatic patients, in asymptomatic patients, the case should be discussed regularly taking into account other parameters as illustrated in the European Society of Cardiology guidelines on congenital heart disease in adulthood [20]. The presence of valvular regurgitation is associated with delayed intrauterine growth and low birth weight (<10th percentile) [21].

Residual stenosis is even worse tolerated than pulmonary insufficiency, whilst mild stenosis is generally well tolerated; it should however be taken into account that the condition will tend to worsen during pregnancy due to the higher volume of circulating blood [20].

Complications of maternal heart disease have been described in 7–10% of cases, largely represented by supraventricular arrhythmias, symptomatic right heart failure, pulmonary embolism and progressive right ventricle enlargement [21].

Frequency of check-ups should be based on the patient’s clinical and echocardiographic status.

Patients receiving treatment with ace inhibitors should suspend treatment due to the risk of teratogenic effects on the foetus, whilst diuretics (furosemide) may continue to be used [14].

Obstetric complications may arise in up to 30% of cases [22]. Vaginal delivery should be preferred, although caesarean section is recommended in the presence of ventricular dysfunction [14].

### 2.4. Ebstein Anomaly

Ebstein anomaly, a moderately complex congenital heart disorder, accounts for less than 1% of all congenital heart diseases [17]. Due to tricuspid valve dislocation, the anomaly is invariably associated with some degree of valvular insufficiency, ranging from minimal to severe depending on the degree of dysplasia of the tricuspid valve [23].

The main problems related to Ebstein anomaly include tricuspid valve insufficiency, heart failure, onset of arrhythmias and cyanosis which are variably manifested due to persistent presence of atrial septal defect or patent foramen ovale with consequent right-left shunt of desaturated blood. Patients displaying saturation levels of <85% and/or heart failure should be advised not to embark on pregnancy (Class IIa Level C) [24].

Patients with valvular insufficiency and/or mild ventricular dysfunction should tolerate pregnancy well. Conversely, patients with significant pre-existing right or left cardiac dysfunction will tolerate pregnancy poorly due to inability of the heart to meet the required increase in cardiac output. Patients with severe symptomatic valvular insufficiency should undergo surgical correction prior to initiating pregnancy [20].

The onset of prevalently supraventricular arrhythmias (including accessory pathway tachycardias such as Wolff–Parkinson–White syndrome) represents the most frequent complication linked to this condition and may result in less positive outcomes [25]. To this regard, women should be trained in the use of vagal manoeuvres (e.g., Valsalva) to disrupt arrhythmia, and taught to recognise the possible early signs of heart failure. However, a series of signs indicative of heart failure may also be routinely observed during pregnancy (e.g., peripheral oedema). Arrhythmias and heart failure give rise to complications in approximately 4% and 3% of pregnancies, respectively [11].

Electrical cardioversion is recommended in hemodynamically unstable patients, whilst those who are hemodynamically stable may be treated by means of vagal manoeuvres or use of adenosine. In patients with broad complex tachycardia, accessory pathway tachycardia cannot be excluded, thus indicating use of flecainide as a substitute for AV nodal blocking drugs [26].

Major risk factors for the foetus are represented by maternal heart failure and degree of cyanosis, resulting in potential premature birth and small for gestational age infants. One study observed lower birth weight in infants delivered to mothers with cyanotic Ebstein anomaly compared to those born to non-cyanotic mothers (2.5 Kg vs. 3.1 Kg) [26]. A perinatal mortality rate of approximately 2% is reported [27].

Frequency of follow-up during pregnancy is guided by the clinical conditions of the mother. Women who are cyanotic women or present with ventricular dysfunction should be seen every one or two months. Vaginal delivery is recommended [24].

### 2.5. Systemic Right Ventricle

Patients affected by systemic right ventricle were born with a transposition of the great arteries which was either congenitally corrected (cc-TGA) or underwent correction by means of palliative surgery. The latter is the case of a dextro-transposition of the great arteries (d-TGA) corrected by means of atrial switch using the Mustard or Senning procedure (involving physiological, but not anatomical, correction of the defect) [18]. Although both defects are described together, it should be highlighted that they are actually two distinct conditions. Systemic right ventricle is graded as CHD of great complexity [17].

Patients with cc-TGA manifest complications associated with dysfunction of the right atrioventricular valve, dysfunction of the systemic right ventricle and electrical conduction disorders leading to complete AV block in 30% of cases [28].

Conversely, the most frequently observed complications in d-TGV patients following atrial switch include arrhythmias (particularly atypical atrial flutter and sinoatrial dysfunction), progressive subaortic left ventricular dysfunction, tricuspid valve insufficiency and intra-atrial baffle obstruction or leaks [29].

**Figure 2:**
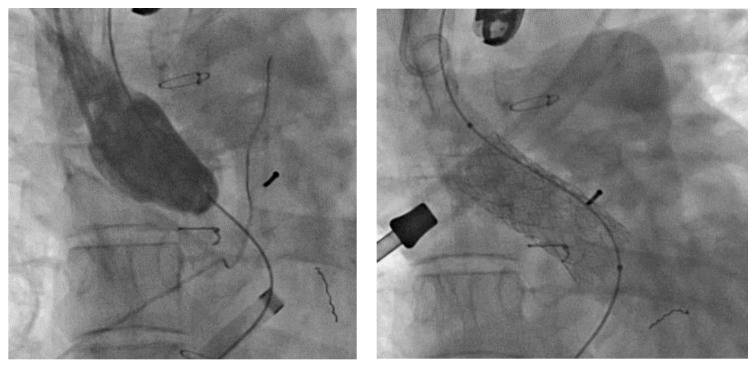
On the (**left**), severe obstruction of systemic venous baffle. On the (**right**), resolution of stenosis with stent implantation.

Accordingly, following a Mustard/Senning procedure, patients with cc-TGA or d-TGA are advised to actively prevent pregnancy, particularly in the presence of an NYHA functional class III–IV, moderate-severe ventricular dysfunction with EF < 40% or severe tricuspid insufficiency (Class IIa Level c) [24]. Likewise, patients awaiting resolution of stenosis of systemic/pulmonary venous baffle are also advised to actively prevent pregnancy (Figure 2).

Literature data relating to post-atrial switch patients are lacking and frequently contrasting. A study published several years ago compared a group of female patients who had undergone palliative atrial switch and later initiated a pregnancy with others who had never been pregnant. Mean duration of follow-up was 100 months. Worsening of systemic tricuspid valve regurgitation was observed amongst the group of pregnant women (52%), whilst no such findings were reported in patients from the other group. However, no significant differences were found with regard to worsening of systolic function, onset of arrhythmias or thromboembolic events. Infants were characterised by lower birth weight and intrauterine growth delay. In more than one third of women, tricuspid insufficiency did not decrease over the post-partum follow-up period [30]. Conversely, other studies also reported deterioration of NYHA functional class, systolic function and baffle obstruction during pregnancy [31,32,33].

The onset of arrhythmias is generally treated with beta-blockers; however, particular caution should be exercised in patients with cc-TGA in view of the tendency to develop bradycardia caused by sinus node dysfunction during atrial switch or atrioventricular block [27].

There is a paucity of data in literature focused on pregnancy in women with cc-TGA, the majority of which based on small retrospective samples. A study conducted by Kowalik et al. of 20 pregnant women with cc-TGA demonstrated that full-term pregnancy can be achieved. The most common cardiovascular complications observed were supraventricular arrhythmias. Long-term follow-up revealed no significant effect of pregnancy on systemic left ventricular function [34]. Presence of cyanosis was associated with a higher rate of spontaneous miscarriage, underlining how maternal hypoxemia represents a significant risk factor for spontaneous miscarriage [35].

Moreover, in pregnant cc-TGA patients, progressive systemic tricuspid valve regurgitation is more severe than that observed following atrial switch in d-TGV [36].

All studies highlighted how patients with systemic right ventricle, either congenital or manifested consequent to palliative surgery, tended to give birth to low birth weight infants [37]. These patients should be directed to tertiary care centres for regular clinical and echocardiographic check-ups, even on a monthly basis where necessary, to detect early signs of heart failure. In both cases, vaginal delivery should be preferred over caesarean section [24].

### 2.6. Pulmonary Hypertension

Pulmonary hypertension (PH) is a disorder manifested secondary to numerous pathological conditions, including congenital heart disease. PH is defined as an increase in mean pulmonary pressure > 20 mmHg, increased pulmonary vascular resistance ≥ 3 Woods units and a wedge pressure ≤ 15 mmHg [38,39].

An additional sub-classification is present in the context of pulmonary hypertension manifested as a consequence of congenital defects [40].

The first group is comprised of patients with Eisenmenger syndrome. Specifically, this includes all intracardiac and extracardiac defects initially manifested as systemic pulmonary shunt and progressing over time to determine severe elevation of pulmonary vascular resistance, thus resulting in systemic-pulmonary or bidirectional shunt.

The second group includes patients affected by arterial pulmonary hypertension associated with prevalently systemic-pulmonary shunt, irrespective of correctability. These defects are of moderate-large entity; vascular resistance is only slightly or moderately increased, although systemic-pulmonary shunt is ongoing and cyanosis is present only on exertion.

The third group is comprised of patients with marked increase in pulmonary vascular resistance in the presence of minor defects (VSD < 1 cm and ASD < 2 cm), or defects detected incidentally which alone would not determine an increase in pulmonary vascular resistance. Closure of these defects is contraindicated.

Lastly, the fourth group includes patients prone to developing pulmonary arterial hypertension either immediately following correction of congenital heart defects or months or even years after correction [40].

In view of the significantly higher risk of maternal mortality both during and after pregnancy in women with pulmonary arterial hypertension, pregnancy is strongly contraindicated (Class III Level B) [24]. The leading causes of death include heart failure, pulmonary embolism, cardiac arrest, uncontrollable bleeding, and endocarditis. Accordingly, these patients are classified as belonging to mWHO group IV, i.e., those featuring an unacceptably high risk [24]. Maternal mortality amongst patients with Eisenmenger syndrome is extremely high, reaching rates of approximately 30% [41].

Neonatal outcome is likewise poor. Foetal and/or neonatal mortality is in the range of approx. 7%, with premature birth occurring in approximately 86% of cases, and failure to thrive in 24% [42].

Taking into account the pulmonary barrage manifested in patients with pulmonary arterial hypertension, it is clearly evident that this condition fails to adapt to the increased blood volume typically observed in pregnancy. Pulmonary circulation is unable to cope with the increased output that impinges on the right ventricle, thus resulting in heart failure. Moreover, these patients already present with an underlying prothrombotic status which is inevitably exacerbated during pregnancy; therefore, patients with Eisenmenger syndrome are characterised by an increased risk of paradoxical embolism. The efficacy of standard pharmacological treatment for PDE during pregnancy remains to be clarified. Advanced pharmacological treatment should be established as early as possible to prevent onset of cardiopulmonary failure. Animal studies have demonstrated the teratogenicity of endothelin receptor antagonists (bosentan, macitentan, and ambrisentan); therefore, other classes of drugs such as phosphodiesterase inhibitors (sildenafil) and/or prostacyclin analogues should be used on a personalised basis. Nifedipine appears however to be safe for use in the treatment of reactive pulmonary arterial hypertension [24,42].

Once the patient has been warned that pregnancy is strongly contraindicated, if she still wishes to face the challenge involved, she should be immediately referred to a tertiary care centre for frequent clinical check-ups (even weekly) and taught how to recognise the early clinical signs and symptoms of heart failure [24].

Delivery will be performed by scheduled caesarean section between 32 and 34 weeks gestation [43,44]. As stated previously, in view of the high mortality rate observed also during the post-partum period, the patient should remain under observation in hospital for up to 2 weeks following delivery [24].

### 2.7. Fontan-Type Univentricular Circulation

This condition is classified as a highly complex heart disease [17] and is associated with a higher risk of infertility, although with the correct management it may be possible to carry pregnancy to term [45]. Appropriate counselling in a tertiary care centre should be provided to assist the patient in planning her pregnancy [24].

This type of circulation is characterised by a higher incidence of arrhythmias, increased risk of thrombosis and high systemic venous pressure. Furthermore, these patients are increasingly prone to developing long-term complications such as protein-losing enteropathy, plastic bronchitis, and systolic and diastolic dysfunction of the single ventricle [46]. As mentioned previously, given the changes that occur routinely during pregnancy, it is evident that in these patients pregnancy is classified as high risk (mWHO classes III and IV) for both the mother and the foetus [24]. Higher rates of miscarriage (up to 70%) and arrhythmias are registered [47]. Hemodynamically significant arrhythmias should be treated by means of urgent electrical cardioversion [26]. Moreover, the foetus may be characterised by intrauterine growth deficits, premature birth and/or neonatal death. In the presence of a cyanotic mother, this risk is even higher. The risk of foetal distress may be linked to premature rupture of the membranes or inability of the Fontan system to adapt to the increased demand for blood by the foetus [10,11,48,49].

There is a relatively low risk (approximately 6%) of the foetus being born with congenital heart disease, although this percentage is higher than that observed in the general population. It is crucial therefore that the mother is given the opportunity to undergo a complete foetal echocardiogram during pregnancy [7].

The increased blood volume typically observed during pregnancy represents a major challenge for a univentricular heart, particularly when dysfunctional or accompanied by insufficiency of the sole atrioventricular valve [50]. To date, no reliable scoring system capable of distinguishing between high risk pregnant women with Fontan circulation and those at relatively lower risk is available. Nonetheless, pregnancy is contraindicated in patients with saturation levels below 85%, dysfunctional single ventricle, moderate/severe insufficiency of the atrioventricular valve, protein-losing enteropathy and relapsing arrhythmias [24].

Indeed, in view of the increased risk of thromboembolism, if not already established, all patients should be prescribed anticoagulant treatment (class IIa) [14,24].

Monthly check-ups should be scheduled both throughout pregnancy and following delivery. Vaginal delivery is preferred due to the lower risk of associated infection, lower blood loss and lower risk of thromboembolism. Women affected by severe heart failure should however be delivered by caesarean section to minimise the effects of continual Valsalva manoeuvres that may be required during vaginal delivery [24].

### 2.8. Mechanical Valves

In women with mechanical valves, pregnancy is linked with a very high -risk of maternal, obstetric, and offspring complications (mWHO class III). In the ROPAC registry, the chances of an event-free pregnancy with a live birth were just 58% for women with a mechanical valve [51].

Regarding maternal risk, the chance of mechanical valve thrombosis is significantly during pregnancy. It decreases with adequate dosing of anticoagulant therapy, varies on the basis of type and position of the mechanical valve, and on the basis of additional patient-related risk factors [52]. Current scientific evidence suggests that the use of warfarin and coumadin during pregnancy -under strict INR control- is the safest way to prevent valve thrombosis from occurring. However, case–control randomised trials in the field are lacking [51]. Low-molecular-weight heparin is probably superior to unfractionated heparin in preventing mechanical valve thrombosis [51].

As regards obstetric and offspring risk, all anticoagulant therapies carry an increased risk of miscarriage and bleeding [51]. In the trade-off between different therapeutic strategies, the ROPAC displays that warfarin and coumadin taken during the first trimester of pregnancy are related to an increased risk of miscarriage compared with low-molecular-weight heparin or unfractionated heparin (28.6% vs. 9.2%) [51].

Oral vitamin k antagonists are the most efficient anticoagulant therapy against these complications in ACHD pregnant ladies. However, they can trigger foetal complications. In fact, since warfarin has a low molecular weight, it crosses the placenta and harms the foetus. It is the so-called foetal warfarin syndrome or warfarin embriopathy, a well-recognised complication of first trimester warfarin use in pregnancy. The disease is characterised by nasal hypoplasia and skeletal abnormalities, including short limbs and digits, and stippled epiphyses. Even when taken in the second or third trimester, warfarin can cause optic nerve atrophy, developmental delay, and microcephaly. ESC guidelines suggest administering warfarin just in the second and third trimester [14]. Maternal and foetal complications are minimised when warfarin dose is ≤5 mg [53,54].

With oral vitamin k antagonists therapy, haemorragic risk during vaginal delivery is not increased. On the contrary, it is augmented in case of caesarian section [7]. Warfarin is not excreted with milk, thereby breastfeeding is not risky [7].

### 2.9. Using Cardiovascular Drugs during Pregnancy

Many cardiac medicines appear safe for use amid and after pregnancy. However, evidence is lacking for some of them. Many of them carry risk of adverse events. Shared decision making, considering risks and benefits of drugs, is crucial to help women weigh up potential risks to themselves and their unborn babies.

Regarding beta blockers, they are commonly used by pregnant women. However, foetal bradycardia, hypotension, and hypoglycaemia are often reported along with low birth weight [55].

Concerning antiarrhythmic agents, amiodarone might be used in special circumstances, although it is linked with a risk of hypothyroidism, goitre, bradycardia, foetal growth restriction, and prematurity at birth [55]. The evidence about the other antiarrhythmic drugs is limited [55].

Diuretics such as furosemide, bumetanide, and hydrochlorothiazide can cause oligohydroamnios and electrolyte imbalance in the foetus.

As regards anticoagulants, warfarin carries a risk of foetal abnormalities if used in the first trimester of pregnancy. It can cause intracranial foetal bleeding in the second trimester. Low-molecular-weight heparin can seldom trigger osteoporosis and markedly less throbocytopenia than unfractionated heparin [55].

The drugs which are contraindicated in pregnancy are angiotensin converting enzyme inhibitors and angiotensin receptor blockers (owing to high risk of foetal abnormalities) and spironolactone (for possible risk of abnormalities of the external genitalia) [55].

### 2.10. Looking Ahead, the Outlook on Personalised Risk Stratification

The risk of CHD recurring in the offspring of women affected by congenital heart defects however is not trivial. As a general rule, foetal echocardiography is routinely used to check the developing heart to rule out this risk, although the presence of CHD may at times be missed at ultrasound scan. In this setting, metabolomics might represent a novel technique for use in detecting CHD in utero with a higher sensitivity and specificity than foetal echocardiography. As an example, several studies have succeeded in identifying different metabolic fingerprints when analysing maternal fluids (amniotic fluid, urine, blood) during the first, second, and third trimester of pregnancy. The findings obtained indicated an apparent link with risk of developing CHD in the offspring [56,57,58,59,60,61]. Overall, these findings provide another step forward in the development of a personalised medicine based on the use of biomarker information in evaluating the treatment options available for any given patient.

## 3. Conclusions

Although not completely devoid of risk, pregnancy can be managed in virtually all patients affected by even the most complex forms of congenital heart disease. It is not however advisable in patients with any form of pulmonary arterial hypertension. Pregnancy is even manageable in patients with Fontan circulation. A personalised risk stratification should be performed, and patients affected by advanced NYHA functional class appropriately warned of the potential risks.

All pregnancies, particularly those at higher risk, should be managed in a tertiary care centre capable of providing the necessary assistance to both the mother and infant.

With a few rare exceptions, vaginal delivery is to be preferred over caesarean section due to the lower degree of maternal and foetal complications (reduced risk of haemorrhage, infections, hemodynamic changes, and risk of coagulation complications).

The desire for motherhood, at times extreme in women with congenital heart disease, may often be accomplished, thus providing a ray of hope in the lives of these patients.

## Figures and Tables

**Table 1:** Pre-, during, and post-pregnancy management according to CHD classification.

Pathophysiology	Specific Congenital Heart Disease	Pre-Pregnancy Management	During Pregnancy Management	Post-Pregnancy Management
1. CHD with increased pulmonary blood flow (septal defects without pulmonary obstruction and with left-to-right shunts)	Scimitar syndrome, interatrial septal defect, complete atrio-ventricular defect, ventricular septal defect, truncus arteriosus, aorto-pulmonary window, patent ductus arteriosus	Counselling (generally mWHO I, unless there is moderate mitral stenosis or post-surgical left ventricular impairment with ejection fraction 30–45% (mWHO III) or severe < 30% (mWHO IV). If truncal valve insufficiency is corrected with a mechanical valve, mWHO risk class is III)	1–2 examinations/9 months for mWHO I; monthly or bimonthly examination for mWHO III; termination or monthly/bimonthly examination for mWHO IV	Discontinuation of heparin after 6 weeks from delivery in those with residual atrial septal defects. Six weeks post-delivery follow-up in mWHO III and IV
2. CHD with decreased pulmonary flow (septal defects with pulmonary obstruction and with right-to-left shunt)	Pulmonary valve stenosis with atrial septal defect, pulmonary stenosis with ventricular septal defect (Tetralogy of Fallot), tricuspid atresia, Ebstein anomaly, single (double inlet) ventricle with pulmonary stenosis	Counselling (mWHO II–IV for Tetralogy of Fallot; mWHO II–III for Ebstein anomaly; mWHO III–IV for single ventricle corrected according to Fontan)	Examination every three months for mWHO II; monthly or bimonthly examination for mWHO III; termination or monthly/bimonthly examination for mWHO IV	Six weeks post-delivery follow-up in mWHO III and IV
3. CHD with obstruction to blood progression and no septal defects (no shunt)	Pulmonary stenosis, aortic stenosis, coarctation of the aorta (adult type)	Counselling (mWHO II-III for severe pulmonary stenosis; mWHO III for asymptomatic severe aortic stenosis; mWHO IV for symptomatic severe aortic stenosis)	Examination every three months for mWHO II; monthly or bimonthly examination for mWHO III; termination or monthly/bimonthly examination for mWHO IV	Six weeks post-delivery follow-up in mWHO III and IV
4. CHD so severe as to be incompatible with postnatal blood circulation	Ductus dependent CHD (pulmonary atresia, aortic and mitral severe stenosis/atresia, aortic arch obstruction), parallel and pulmonary circulations (complete transposition of the great vessels), anomalous connection/obstruction of the pulmonary veins (total anomalous pulmonary venous drainage, cor triatriatum sinister)	Counselling (mWHO II–IV for pulmonary atresia; mWHO IV for severe mitral stenosis; mWHO II–IV depending on the degree of aortic coarctation; mWHO I for transposition of the great vessels corrected with arterial switch; mWHO III–IV for transposition of the great vessels corrected with atrial switch	1–2 examinations/9 months for mWHO I; monthly or bimonthly examination for mWHO III; termination or monthly/bimonthly examination for mWHO IV	Six weeks post-delivery follow-up in mWHO III and IV
5. CHD silent until adult age	Bicuspid aortic valve, congenitally corrected transposition of the great vessels	Counselling (mWHO III for bicuspid aortic valve with moderate aortic dilatation or congenitally corrected transposition of the great vessels with good or mildly decreased right ventricular function; mWHO IV for bicuspid aortic valve with severe aortic dilatation or congenitally corrected transposition of the great vessels with moderately or severely decreased right ventricular function	Monthly or bimonthly examination for mWHO III; termination or monthly/bimonthly examination for mWHO IV	Six weeks post-delivery follow-up in mWHO III and IV

## Data Availability

Not applicable.
